# Discovering transnosological molecular basis of human brain diseases using biclustering analysis of integrated gene expression data

**DOI:** 10.1186/1472-6947-15-S1-S7

**Published:** 2015-05-20

**Authors:** Kihoon Cha, Taeho Hwang, Kimin Oh, Gwan-Su Yi

**Affiliations:** 1 Department of Bio and Brain Engineering, KAIST, Daejeon 305-701, South Korea; 2 Department of Information and Communications Engineering, KAIST, Daejeon 305-701, South Korea

## Abstract

**Background:**

It has been reported that several brain diseases can be treated as transnosological manner implicating possible common molecular basis under those diseases. However, molecular level commonality among those brain diseases has been largely unexplored. Gene expression analyses of human brain have been used to find genes associated with brain diseases but most of those studies were restricted either to an individual disease or to a couple of diseases. In addition, identifying significant genes in such brain diseases mostly failed when it used typical methods depending on differentially expressed genes.

**Results:**

In this study, we used a correlation-based biclustering approach to find coexpressed gene sets in five neurodegenerative diseases and three psychiatric disorders. By using biclustering analysis, we could efficiently and fairly identified various gene sets expressed specifically in both single and multiple brain diseases. We could find 4,307 gene sets correlatively expressed in multiple brain diseases and 3,409 gene sets exclusively specified in individual brain diseases. The function enrichment analysis of those gene sets showed many new possible functional bases as well as neurological processes that are common or specific for those eight diseases.

**Conclusions:**

This study introduces possible common molecular bases for several brain diseases, which open the opportunity to clarify the transnosological perspective assumed in brain diseases. It also showed the advantages of correlation-based biclustering analysis and accompanying function enrichment analysis for gene expression data in this type of investigation.

## Background

It has been shown that various neurodegenerative diseases and even psychiatric disorders have a large extent of similarity in terms of neurological dysfunction [1]. In clinical practices in these diseases, transnosologically oriented treatments are often used based on the assumption of this similarity. Therefore, it seems important to find common molecular mechanisms of transnosological aspect of neurological diseases as well as the unique characteristics of individual diseases. The similarity among some representative brain diseases has been observed and summarized at the phenotypic level. However, the molecular level commonality is yet largely uncharacterized due to the lack of the application of currently developed method appropriate for this type of investigation.

Transcriptome analysis have been suggested for comprehensive gene level interpretation of many diseases. Transcriptome data is also one of the most available resources for human brain study. Various methods for transcriptome analysis have been developed for identification of differentially expressed genes, correlated genes, and significant functional modules [2-7]. There have been several transcriptome analyses of human brain diseases using microarray [8]. Most of the previous studies focused on identifying differentially expressed genes under various neurodegenerative diseases such as Alzheimer's disease [9-11]. Though they suggested sets of individual molecular players associated with brain disease etiology, it has been recommended to investigate system-level transcriptomic changes by identifying gene sets that show coexpression under either brain disease or normal condition in order to get more comprehensive understanding on highly complex brain diseases [8,12]. Those previous approaches were, however, restricted to individual brain diseases so that identification of molecular basis common to multiple brain diseases was difficult. Integrative analysis uses an integrated data of multiple microarray datasets via adjusting batch effects, or systematic difference between datasets, which is promising approach to identify robust molecular basis associated to multiple brain diseases. In addition, biclustering analysis is a good candidate for searching coexpressed gene sets under specific subset of samples. Given that there are many brain diseases to be simultaneously investigated and it is unclear which brain disease combinations would have common molecular basis, a biclustering analysis is expected to provide efficient method to identify common coexpressed gene sets under various combinations of brain diseases. Both integrative approach and biclustering analysis showed its utility in studies of other transcriptome data for complex diseases such as cancers [13,14] but there have not been applied to investigate brain transcriptome data.

To this end, we used an integrative approach on three microarray datasets covering five neurodegenerative diseases, three psychiatric disorders and normal conditions to identify common molecular basis of various brain disease combinations in a transnosological manner. Using a biclustering analysis and post-processing, we efficiently identified large number of gene sets that gained or lost correlation under all or specific combinations of brain diseases compared to the normal conditions. Large portion of those common gene sets were enriched for genes having known association with brain diseases, implicating system-level change of transcriptional organization to the multiple brain diseases. A function enrichment analysis on the identified gene sets showed that the common coexpressed gene sets were more enriched for the biological processes associated with neurological processes compared to individual single disease-specific gene sets, indicating that they might serve as good starting points in studies discovering key targets of drug intervention of transnosological aspect.

## Results

### Identification of brain disease-specific coexpressed gene sets

We aimed to identify coexpressed gene sets either in brain diseases or in normal to find molecular mechanisms potentially associated with brain diseases. Especially, we focused on finding molecular mechanisms common to multiple brain diseases. We chose five neurodegenerative and three psychiatric disorders, covering Alzheimer's disease, Parkinson's disease, Huntington's disease, multiple sclerosis, amyotrophic lateral sclerosis, schizophrenia, bipolar disorder, and autism along with controls. For an integrative gene expression analysis, we combined three microarray datasets into a single dataset by adjusting batch-specific effects using ComBat method [15]. As a result, we could get a microarray dataset with 6688 distinct genes and 237 samples. We applied the biclustering method to the combined microarray dataset to efficiently get initial sets of biclusters in which at least genes show correlated expression values under arbitrary subset of samples with average Pearson's correlation coefficients (PCC) equal or greater than 0.7 in a transnosological manner. We chose 0.7 as the threshold based on our empirical simulation result that the probability that an arbitrarily composed bicluster has average PCC equal or greater than 0.7 is statistically significantly rare (P = 1E-04). We selected only those biclusters containing all samples from one or more of classes (e.g. all samples from multiple sclerosis class and all samples from Alzheimer's disease class). We eliminated samples from each of the selected biclusters if the samples are just part of certain class. With the refined biclusters, we next determined whether the gene coexpression is substantially gained or lost in brain diseases compared to normal. For this process, we separately calculated average PCC of the genes included in each refined bicluster in included brain diseases and in normal, and got the difference in the average PCCs. We assigned p-values to the difference by using background distribution of difference of average PCCs between brain diseases and normal from 100,000 random gene groups. We finally identified coexpressed gene sets showing statistically significant gain or loss of coexpression in brain.

**Figure 1:**
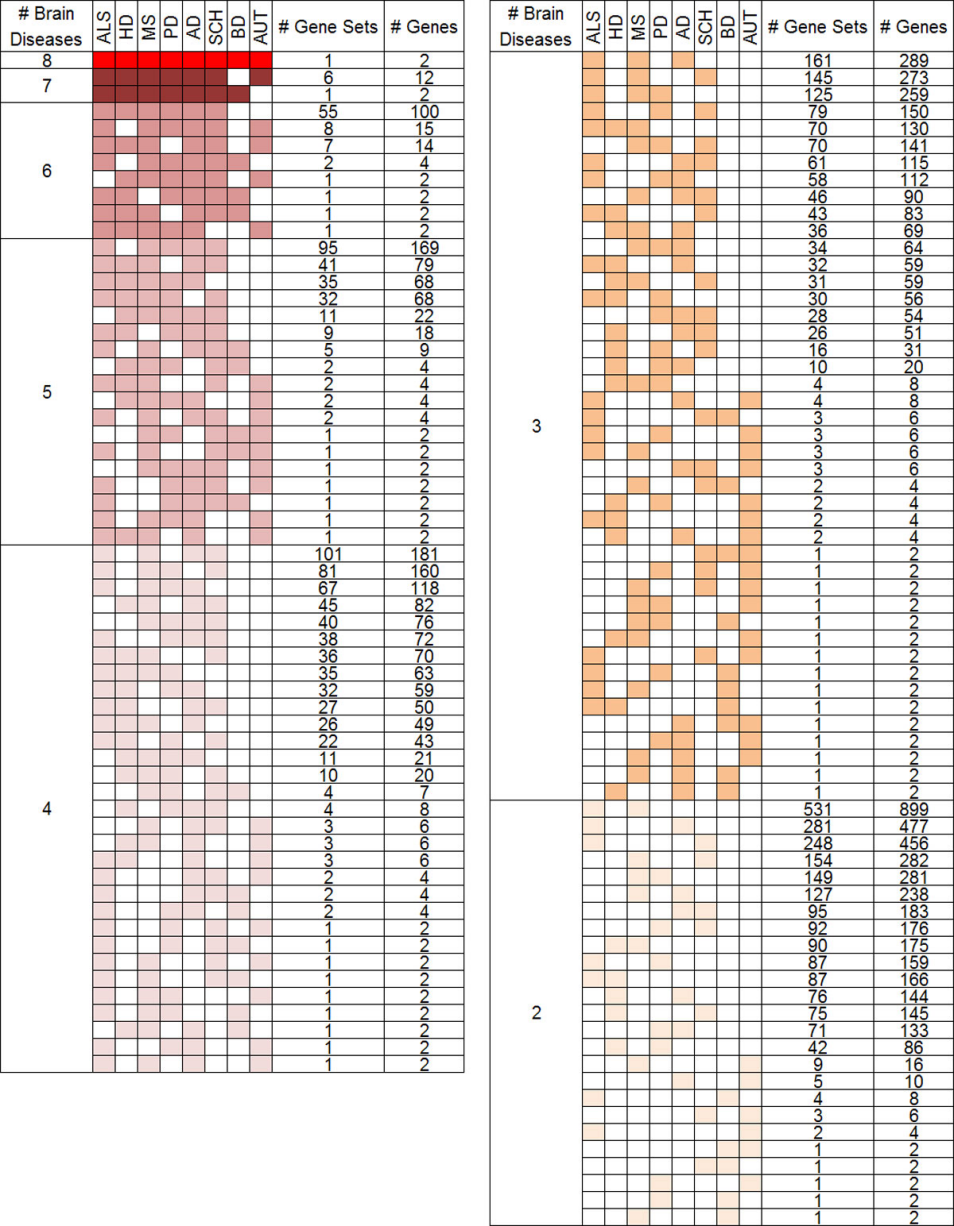
**Summary of brain disease combinations sharing common coexpressed gene sets**.

A total of 4,307 gene sets were identified to be coexpressed in at least two brain diseases, implying that there might be large number of molecular mechanisms commonly associated to multiple brain diseases with Benjamini corrected p-value of 0.005. Given that the number of coexpressed gene sets specific to individual brain diseases is 3,409, our finding supports the hypothesis that there are huge similarity between the investigated brain diseases at molecular level for the first time. The numbers of shared coexpressed sets show degree of association among different combinations of brain diseases in Figure 1. We found the similarity group among brain diseases by the number of total coexpressed gene sets between all possible combinations of two brain diseases. ALS and MS is the most similar disease with 1,684 coexpression gene sets. Other brain diseases are listed with AD, SCH, PD, HD, AUT, and BD in the order of the number of shared gene sets. This result shows shared mechanism of molecular level among neurodegenerative diseases. Particularly, SCH, classified by psychiatric disorder, highly correlated with neurodegenerative disease.

**Figure 2:**
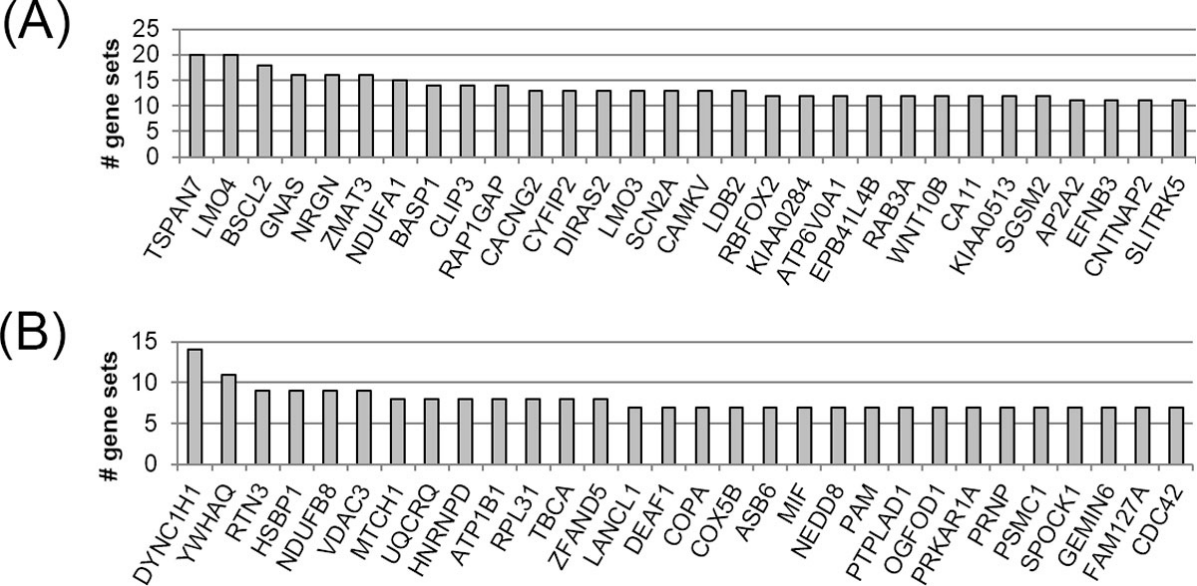
**Top 30 genes most frequently found in the coexpressed gene sets**. (A) Gene sets shared by at least two brain diseases and (B) single brain disease-specific gene sets.

Figure 2 shows two types of top 30 genes in gene sets of single brain disease and at least two brain diseases. To discover the biological meaning and functions of each gene set from single and multiple brain disease, function enrichment analysis of each gene list was performed using DAVID. As the results, the fourteen genes (RTN3, LANCL1, YWHAQ, COX5B, DYNC1H1, HNRNPD, MIF, NEDD8, PRKAR1A, PSMC1, RPL31, TBCA, VDAC3, and CDC42) in gene sets of one brain disease were significantly enriched only 'acetylation' term of SwissProt PIR Keyword with Benjamini corrected p-value of 0.006. The three genes (AP2A2, CACNG2, and RAB3A) in gene sets of at least two brain diseases were significantly enriched 'synaptic transmission' term of Reactome pathway with Benjamini corrected p-value of 0.022. This result shows that frequently found genes in gene sets sharing brain diseases are different from genes of only one brain disease in terms of cellular function. In particular, frequently observed genes in gene sets sharing multiple brain diseases are distributed the important pathway of neurological processes.

### Coexpressed gene sets across multiple brain diseases are enriched for known disease-associated genes

We could find 1 and 7 coexpressed gene sets shared by all and 7 different brain diseases, respectively in Table 1. To find association between the genes in 8 coexpressed sets and brain diseases, we checked genes in coexpressed gene sets with known brain disease-associated genes by directly and first-order interacting proteins. First, we collected brain disease-associated genes using public available disease databases. The collected 2,697 genes comprising 1,310 for AD, 985 for SCH, 534 for MS, 517 for BD, 352 for PD, 186 for AUT and 44 for HD are used. Second, we used our comprehensive protein interaction database, ComBiCom [16], to find nonredundant protein interaction relationships. As the result, the three genes (ATP1A1 for BD, RBFOX1 for BD, and KLC1 for AD and PD) are directly related to brain diseases. Moreover, 10 genes (ATP1A1, ATP6V1D, C16orf45, CROCC, KLC1, LUC7L2, PIAS2, PRKAR1B, RBFOX1, and RPL19) interacts with at least one brain disease-associated gene. Our approach offers the potential to discovery more reliable and accurate drug targets covering multiple brain diseases with shared molecular mechanisms. These genes are also associated with brain function, biological process or pathway, such as hormone synthesis, metabolic pathway, cell death, synaptic vesicle cycle, and nervous system development.

**Table 1:** Coexpressed gene sets shared by more than 7 brain diseases.

Brain diseases sharing gene sets								Avg. PCC		Diff significance*		Genes
**AD**	**ALS**	**HD**	**MS**	**PD**	**SCH**	**BD**	**AUT**	**Brain disease**	**Normal**	**P-value**	**Benjamini**	

1	1	1	1	1	1	1	1	0.908	0.509	1.30E-03	4.804E-03	AKR1D1, FKTN
1	1	1	1	1	1	0	1	0.854	0.084	4.00E-05	8.759E-04	LUC7L2, SULT1A1
1	1	1	1	1	1	0	1	0.884	0.343	4.20E-04	2.791E-03	ARHGEF3, ATP1A1
1	1	1	1	1	1	0	1	0.711	0.184	4.70E-04	2.977E-03	CROCC, PDE9A
1	1	1	1	1	1	0	1	0.879	0.431	9.60E-04	4.301E-03	C16orf45, RBFOX1
1	1	1	1	1	1	0	1	0.813	0.368	9.80E-04	4.301E-03	PIAS2, PRKAR1B
1	1	1	1	1	1	0	1	0.820	0.417	1.27E-03	4.790E-03	RPL19, RPL39
1	1	1	1	1	1	1	0	0.800	0.404	1.32E-03	4.828E-03	ATP6V1D, KLC1

* Diff. significance: Statistical significance of the avg. PCC brain disease - avg. PCC normal empirically calculated based on the background distribution of 100,000 random gene groups.

### Functional characteristics of coexpressed gene sets shared by multiple brain diseases

**Figure 3:**
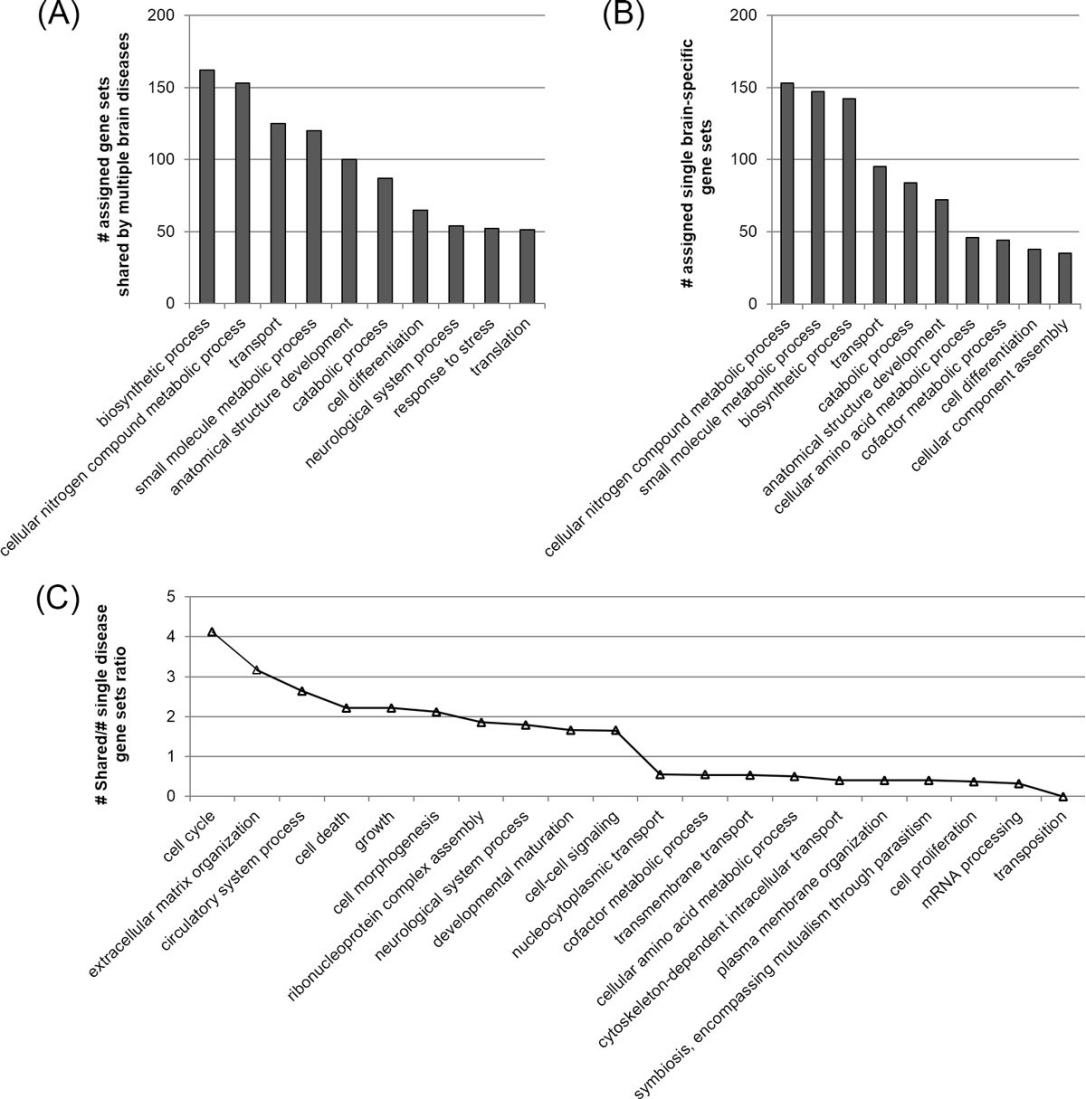
**Functional distribution of the shared gene sets and the single brain disease-specific gene sets**. The x-axis shows the representative functional categories (biological processes) selected. (A)(B) The number of assigned gene sets shared by multiple brain disease and single brain-specific gene sets in each functional category. (C) Ratio of the number of shared gene sets over single disease-specific gene sets

As we observed previously, there might be functional difference between the single brain disease-specific gene sets and shared gene sets. We further investigated the functional characteristics of the 4,299 shared gene sets in comparison with the 3,409 disease-specific gene sets. For this, we carried out a function enrichment analysis for each shared gene sets and single disease-specific gene sets using the Gene Ontology (GO) biological process at Benjamini corrected p-value of 0.01. We assigned each gene sets to one or more of the representative biological processes according to the enriched biological processes to identify the functional categories that are relatively overrepresented by the multiple brain disease gene sets compared to the single disease-specific gene sets. For fair comparison, we normalized the number of assigned gene sets by dividing the number of identified gene sets in each functional category by the total number of identified gene sets. Figure 3A and Figure 3B shows 10 functional categories of the highest counts in multiple brain disease-specific and single brain disease-specific gene sets. In Figure 3C, we only showed 20 functional categories showing the highest and the lowest fold. While the single brain disease-specific gene sets were more frequently enriched for the metabolic processes, the functional categories such as "cell cycle", "neurological system process" and "cell-cell signaling" had nearly 2-fold greater representation among the shared gene sets. This is notable since the category "neurological system process" involves a wide variety of biological processes that directly regulate or at least substantially affect neurological processes at the phenotypic level. The overrepresentation of "cell morphogenesis", "cell death", "developmental maturation", and "cell-cell signaling", indicates that the shared gene sets are more likely to have implications for brain cell development and degradation, and neuron-to-neuron or glia-to-neuron interaction, respectively. Taken together, our data suggests that the shared molecular bases among multiple brain diseases are more enriched in the functional categories that are associated with neurological function. This might reflect the fact that the multiple brain diseases in our study have great similarity in terms of neurological deficit even though the pathology or other symptoms vary greatly from disease to disease. Thus, identifying the shared gene sets rather than sing disease-specific gene sets might increase the chances of discovering more extensive and plausible molecular bases that are tightly associated with neurological impairment.

## Conclusions

We carried out an integrative biclustering analysis of transcriptome data from multiple diseases that are known to similarly neurological dysfunction. We demonstrated large number of potential molecular bases shared among multiple brain diseases and revealed high extent of heterogeneity among samples in a disease, which collectively emphasize the benefits of our analysis for the identification of the novel molecular mechanisms underlying multiple brain diseases in a transnosological manner. Our analysis showed that the number of gene sets in multiple brain disease is substantially larger than that of single disease-specific gene sets, and that the shared gene sets are more likely to be associated with the pronounced biological processes underlying cognition. The results provide new type of information on the specific relationships among genes, gene sets, and combinations of brain diseases, which form a complex network that covers several known facts and suggests previously unknown relationships and characteristics. This study can be used as a promising strategy that efficiently maximize utilization of currently limited human brain transcriptome data for the extensive identification of potentially valuable molecular basis for neurological dysfunction commonly in multiple brain diseases.

## Methods

### Collection of microarray data

Microarray data were derived from the NCBI GEO web site by keyword search using the names of the neurodegenerative and psychiatric disorders. Since we are interested in brain mechanisms, we focused only on the microarray data produced using postmortem human brain samples and tried to minimize the number of microarray data to be combined while trying to maximize the number of brain diseases. We selected 3 sets of microarray data: GSE26927, GSE5388, and GSE28521. The set GSE26927 included 11 samples with Alzheimer's disease, 10 samples with amyotrophic lateral sclerosis, 10 samples with Huntington's disease, 10 samples multiple sclerosis, 12 samples with Parkinson's disease, 10 samples with schizophrenia, and 55 control samples extracted from the entorhinal cortex; this dataset is based on the Illumina humanRef-8 v2.0 expression beadchip. The GSE5388 dataset included 30 samples with bipolar disorder and 31 control samples of the dorsolateral prefrontal cortex measured on the Affymetrix Human Genome U133A Array (Affymetrix Santa Clara, CA) [17]. Lastly, the GSE28521 data was for 29 autism patients and 29 controls and was based on the Illumina HumanRef-8 v3.0 expression beadchip [12]. We parsed the SOFT formatted files of 3 sets of microarray data using the GEOquery R package [18].

### Combining microarray data

We transformed the GSE28521 data by inverting the log2-transformed expression data using the exponential function. Then, we applied quantile normalization to each of the 3 selected sets of microarray data using the functionalities in the limma R package [19]. Because all the selected microarray data have different platforms, we converted the probe IDs into Entrez Gene IDs using the biomaRt R package for the GSE5388 dataset and the lumiHumanIDMapping R package for the GSE26927 and GSE28521 data sets in order to allow the different datasets to possess a unified ID structure. After excluding probes whose expression values were missing in GSE28521, we identified probe IDs that were mapped to the same Entrez GeneIDs in each set of microarray data and collapsed their expression values by averaging them to make each microarray dataset contain non-redundant sets of genes. Then, we combined the 3 preprocessed sets of microarray data using ComBat [15], the batch effect adjusting method known to outperform other methods [20] implemented in the inSilicoMerging R package [21].

### Identification of coexpressed gene sets

We used BICLIC, our recently developed biclustering method [5], to efficiently identify sets of genes that are correlated in individual or multiple combinations of non-normal (i.e. brain diseases) in an unsupervised manner. The BICLIC divides the clustering problem into individual sample clustering problems. In each sample, the ordered gene expression values were grouped into small sized clusters to efficiently identify initial seed biclusters by collecting samples in which clusters had the same genes. The seed biclusters had at least 2 genes and 3 samples, in accordance with the minimum requirement for the normal calculation of the Pearson's correlation coefficient (PCC) as coexpression measurement. We expanded each seed bicluster in 2 ways by including genes and samples, while keeping the average PCC of the expanded bicluster that is higher than the designated correlation threshold, 0.7. We set the maximum number of genes in a bicluster at 50 in this analysis, but we did not set the maximum number of samples, in order to focus on the identification of molecular bases shared by as many brain diseases as possible. Once a seed bicluster is expanded in the gene direction and in the sample direction, separately, we can assume that this is a bicluster that contains all of the genes and samples needed to set a search space for identifying an optimal bicluster. Within the search space, we eliminated subsets of genes or samples until the average PCC became larger than the correlation threshold for the first time, while maximizing the number of involved samples.

### Function enrichment analysis

To identify functional meaning of discovered coexpressed gene sets, we performed functional enrichment analysis by COFECO [22]. We selected the biological process of Gene Ontology terms with a corrected p-value of 0.005.

### Collection of brain disease-associated genes

We collected disease-associated genes from six databases: OMIM (Online Mendelian Inheritance in Man), Genetic Association Database [23], PharmGKB [24], KEGG DISEASE [25], and Huge Navigator [26]. Because the disease names vary among the source databases, we standardized the disease names by extracting the Unified Medical Language System (UMLS) [27] IDs using MetaMap [28]. The UMLS IDs were converted once more into ICD-10-CM (International Classification of Diseases, 10th Revision, Clinical Modification) using the mapping information provided in the UMLS.

## List of abbreviations

AD, Alzheimer's disease; HD, Huntington's disease; PD, Parkinson's disease; ALS, amyotrophic lateral sclerosis; MS, multiple sclerosis; SCH, schizophrenia; BD, bipolar disorder; and AUT, autism.

## Competing interests

The authors declare that they have no competing interests.

## Authors' contributions

KC carried out the selection of microarray datasets, biclustering analysis, functional analysis, and drafted the manuscript. TH carried out the preprocessing and integration of microarray dataset. KO participated in the functional interpretations of coexpressed gene sets. GSY conceived of the study, participated in its design and coordination, functional interpretation, and drafted and reviewed the manuscript. All authors read and approved the final manuscript.

## Declaration

Publication costs for the article were sourced from the Bio-Synergy Research Project (NRF-2012M3A9C4048759) of the Ministry of Science and the National Research Foundation of Korea (NRF) grant funded by the Korea government (MSIP) (No.2010-0028631)

This article has been published as part of *BMC Medical Informatics and Decision Making *Volume 15 Supplement 1, 2015: Proceedings of the ACM Eighth International Workshop on Data and Text Mining in Biomedical Informatics. The full contents of the supplement are available online at http://www.biomedcentral.com/bmcmedinformdecismak/supplements/15/S1.
